# A social norms campaign based positive intervention aimed at promoting protective behaviours

**DOI:** 10.3389/fpubh.2024.1447335

**Published:** 2024-10-24

**Authors:** Esther Cuadrado, Alicia Arenas, Carmen Tabernero, Miguel A. Maldonado

**Affiliations:** ^1^Departamento de Psicología, Universidad de Córdoba, Cordoba, Spain; ^2^Instituto Maimónides de Investigación Biomédica de Córdoba (IMIBIC), Cordoba, Spain; ^3^Hospital Universitario Reina Sofía, Cordoba, Spain; ^4^Departamento de Psicología Social, Universidad de Sevilla, Seville, Spain; ^5^Departamento de Psicología Social, Universidad de Salamanca, Salamanca, Spain; ^6^Instituto de Neurociencias de Castilla y León, Salamanca, Spain

**Keywords:** behavioural intention, COVID-19, protective behaviours, self-efficacy, social norms campaigns, experimental design, university students, public health

## Abstract

**Introduction:**

Social norms campaigns are communication strategies designed to influence people’s behaviour by highlighting the social norms of their reference group. Such campaigns have been shown to be effective in promoting healthy behaviours in a variety of settings. This study explored the effectiveness of a social norms campaign applied to COVID-19 protective behaviours among university students during the pandemic.

**Methods:**

A total of 141 university students (83.1% female, 16.9% male) with a mean age of 21.55 years (*SD* = 4.33) initially took part in an experimental pre-test-post-test longitudinal panel study between January and July 2022, with participants randomly assigned to a control group (46 participants) or an experimental group (95 participants). Considering the experimental attrition, only 83 participants completed the last questionnaire (81.9% female, 18.1% male; mean age = 22.12 years, *SD* = 5.29), of whom 32 belonged to the control group and 51 to the experimental group.

**Results:**

The Student’s *t*-test show that participants in the experimental group, who were exposed to the campaign, reported higher levels of self-efficacy, protective behavioural intention, and protective behaviours than the control group.

**Discussion:**

It is concluded that social norms campaigns applied to COVID-19 protective behaviours are effective in times of pandemic and might be extrapolated to other epidemic contexts.

## Introduction

The pandemic situation highlighted that human behaviour plays a crucial role in reducing the spread of infectious diseases. According to the WHO (1), since the appearance of SARS-CoV-2 in December 2019, causing the COVID-19 pandemic, the number of people infected has reached 775 million, of whom approximately 7.1 million around the world have died.

In Spain, according to WHO (1) data and according to the latest report published by the Ministry of Health of the Government of Spain (2), since the beginning of the pandemic, 13.9 million people have been infected and 121,852 have died. Due to the high rate of infection and mortality, governments have promoted different protective measures for people’s health for collectives and individuals. In this context, it has been demonstrated that the use of masks or social distancing significantly reduces the transmission of contagious diseases that are transmitted via respiratory modes (3), these behaviours being influenced by individual, social, and contextual factors (4). Thus, the adoption of protective measures in a health and global pandemic context is essential to limit disease transmission.

However, populations have not always adopted the necessary protective measures during the pandemic. Some risk behaviours for virus transmission have been demonstrated by a small percentage of the population: for instance, some people have remained highly socially active and adopted few protective behaviours (5, 6). In this regard, younger populations, though adopting protective behaviours for the most part, have been shown to be somewhat less likely to do so than older populations (7).

In this context, it seems relevant to analyse the effectiveness of measures to promote the adoption of protective behaviours in younger populations. The present study therefore explored the effectiveness of a social norms campaign derived from the theory of planned behaviour (TPB) (8) applied to protective behaviours in times of pandemic.

### Social norms campaigns as instruments to promote protective behaviours

Social norms campaigns are communication strategies designed with the objective of promoting positive behavioural changes by highlighting the positive social norms of a given social context to raise awareness of what is considered socially acceptable (9). Indeed, Cialdini et al. (9) highlight the importance of social norms in regulating human behaviour. By focusing on how descriptive and injunctive norms affect behaviour and how attention to these norms can be directed, the focused theory of normative behaviour (9) offers possible strategies for positively influencing social behaviour. The theory suggests that behaviours are more likely to be influenced by norms that are the focus of attention in a specific context. For example, when faced with the transmission of a disease, the social norm may be to protect oneself from the disease in order to avoid further contagion and not get sick: there is thus a greater probability that people will protect themselves by following this norm, since it is focused on healthy and protective behaviours against the disease. This mechanism can be useful through the implementation of social norms campaigns that make the social norm visible and give precise information about it. These campaigns are based on the concept of social norms proposed in the TPB (8), a theory that has become key in the understanding and promotion of health behaviours (10, 11), specifically in the promotion of protective behaviour in times of pandemic (7, 12, 13). The TPB describes how behavioural attitudes, subjective social norms, and behavioural control influence people’s behavioural intentions, which in turn influence the enactment of a determined behaviour (14). Specifically, subjective social norms—the central element in social norms campaigns—refer to individuals’ personal perception of the normative behaviour of their reference group, working as a kind of social pressure that will influence final behaviour (8). In other words, the idea underlying a social norms campaign is that people are influenced by their beliefs about what their reference group members do (descriptive norms) or are expected to do (injunctive norms).

However, as discussed in the systematic review by Robinson et al. (15) on eating behaviours and the effect produced by social norms, descriptive and injunctive norms do not have the same effects. Robinson et al. (15) make a conceptual differentiation between injunctive norms and descriptive norms: descriptive norms provide information about what most people do, whereas injunctive norms refer to norms based on social approval, by providing information about what people are expected to do. Therefore, it could be expected that when individuals see that most people in their reference group act or are expected to act properly, then individuals will act properly, influencing behavioural changes in themselves and in society (16).

Nevertheless, Robinson et al. (15) claim that, given that the participants in their study ate alone, it is unlikely that social approval (injunctive norms) guided their behaviour. In fact, there is evidence (15, 17, 18) that descriptive norms (information about others’ consumption of certain foods) significantly influence the selection and ingestion of those same foods, whereas injunctive norms (information about whether others approve of the consumption of those foods) have no effect on behaviour. These same effects on behaviour have been demonstrated in adolescent populations (17) and in young adults (19). Moreover, Schultz et al. (20) found in a social norms campaign experiment that descriptive norms, but not injunctive norms, have a significant impact and long-term effects on people’s behaviour. Thus, the application of social norms campaigns based on descriptive norms has been a useful and effective tool at the level of intervention in health behaviours, and specifically in behaviours such as eating (21).

In this way, descriptive norms influence behaviour by altering the degree to which an individual perceives the behaviour in question to be beneficial to them (e.g., in terms of health) in order to be in line with the social norm and thus adapt their behaviour appropriately to the group. Based on this fact, social norms could be applied to promote protective behaviours against disease, since by informing individuals about what others are doing, they could modify their behaviour to be in line with the social norm and adapt, benefiting both their psychological wellbeing and health.

By using the TPB model in the pandemic context, Cuadrado et al. (7) found that subjective descriptive social norms about protective behaviours significantly influence people’s protective behaviour. Specifically, it has been demonstrated that subjective social norms have a greater effect on protective behaviours in younger people than in older people, by affecting their protective behaviour not only indirectly (as expected in the TPB, and as for older people), but directly, too. According to Cuadrado et al. (7), social norms campaigns aimed at young people should be effective in increasing their protective behaviour against COVID-19, this target group having reported lower adoption of protective behaviour than older people.

Additionally, social norms campaigns have been shown to be effective in different health promotion settings, such as anti-smoking (22) or traffic safety campaigns (23). However, there is currently no evidence for the effectiveness of social norms campaigns specifically regarding COVID-19 protective behaviours. Due to the great potential of social norms as a tool for adjusting behaviour, better understanding of the mechanisms by which social norms work is needed, in particular for the promotion of protective behaviours. Thus, the present study aimed at applying social norms campaigns to protective behaviours against the transmission of COVID-19 in university students, a group that must be a target for this kind of intervention and that is especially influenced by social norms (7).

#### Social norms campaigns as instruments to promote self-efficacy

Another particularly relevant component that social norms campaigns can consider is self-efficacy. One component of the TPB model is behavioural control, which consists of two different dimensions (24): the beliefs individual hold about the controllability of their behaviour, and their belief in their own ability to perform an output behaviour properly and effectively (self-efficacy).

Bandura (25) argued that self-efficacy influences behaviour such that when someone has high self-efficacy they are more likely to perform the behaviour and have greater success, whereas low self-efficacy may limit action in the face of challenges. Indeed, the effect of self-efficacy on the adoption of health protective behaviours has been demonstrated in different studies and settings (26–28), along with its role in predicting protective behaviours in times of pandemic (29–31). In this sense, it would be interesting to explore whether social norms campaigns also influence self-efficacy, which has been shown to act as a predictor of protective behaviours.

Whilst we are not aware of studies analysing the possible relationship between social norms and self-efficacy, it can be inferred from the Bandura (25) own theory that social norms could be a source of self-efficacy. Bandura (25) reveals that vicarious experience is a specific source of self-efficacy. When observing models that are effective in computing a particular behaviour, people can strengthen their belief in their own ability to achieve similar results, thus increasing their self-efficacy in respect of this behaviour. Moreover, for models to be effective, they must be meaningful to the person and be evaluated as having a similar level of ability. Social norms campaigns that expose the positive descriptive social norms of the reference group of individuals (i.e., of a group to which the recipients of the campaign belong, a group whose members the recipient of the campaign evaluates as having a similar level of ability) might therefore act as a sort of vicarious experience that could increase individuals’ self-efficacy; or perceptions of what our reference group members think and do (social norm) could function as a model and increase our belief in our ability to behave like this reference group. Therefore, it is interesting to explore whether social norms campaigns also function as a kind of modelling. If this is the case, social norms campaigns should influence perceived self-efficacy, a predictor of protective behaviours.

#### The present study

Based on evidence of the effectiveness of social norms campaigns, the present study aimed to apply such a campaign to the pandemic context and to the protective behaviours of a young population. Considering that subjective social norms and self-efficacy influence behavioural intention and thus the behaviours themselves, it was expected that applying a descriptive social norms campaign to a group of university students would influence their behavioural intention and behaviour. Moreover, as being exposed to the social norms of one’s reference group during a social norms campaign could act as a kind of modelling, it was expected that applying a descriptive social norms campaign to a group of university students would influence their self-efficacy. Therefore, the following study hypothesis (H) was proposed:

*H1.* Exposure to a social norms campaign applied to university students’ protective behaviours will increase their (H1a) intention for protective behaviours; (H1b) self-efficacy for protective behaviours; and (H1c) protective behaviours.

## Methods

### Procedure

#### Participant recruitment

Participants were recruited from different Spanish universities by non-probabilistic convenience sampling through the university academic platforms used by the professors involved in the study. Information was posted on these platforms requesting the participation of university students for a research project about behaviours and attitudes in relation to the COVID-19 pandemic. Likewise, they were informed that participants who participated in all phases of the study could win a 2-in-1 tablet in a raffle. A total of 900 university students showed interest in participating by providing their mail in an online questionnaire provided in the posted information. Those 900 potential participants were listed in SPSS to assign 750 of them randomly to the first part of the study (application of a questionnaire to obtain information on the objective descriptive social norm concerning university students’ protective behaviours related to COVID-19 to be able to design a social norms campaign), and 150 to the second part of the study (to explore the effectiveness of the designed social norms campaign). Once all the phases of the Study were completed, all the participants were fully debriefed and informed about the study objective and procedure. The text provided in the debriefing can be seen on Supplementary material.

#### First part of study: obtaining the reference group’s objective social norm

Positive objective social norms for the reference group are a necessary precondition for a social norms campaign, because the social norms campaign will result from this information. Therefore (and considering that in this study, the reference group of the recipients of the campaign is university students), prior to the implementation of the social norms campaign in the second part of the study, a questionnaire was sent to the 750 participants who were randomly assigned to the first part of the study to collect information about their COVID-19 protective behaviours. All but one of the recipients completed the questionnaire, giving information about their COVID-19 protective behaviours, from which the university students’ objective social norms could be inferred.

To know what the social norm of university students is, we analyse the percentage of them carrying out protective behaviours. In this way, if what most participants do reflect appropriate protective behaviours against COVID-19, those positive behaviours can be used as information about the group’s positive social norms to encourage the increase in these positive behaviours through a social norms campaign. The data showed positive results, demonstrating that the objective social norm of the surveyed student population was generally positive in relation to protective behaviours, most of them perceiving such protective measures as positive and adopting them in their daily lives. More specifically, the data showed that for the first objective social norm measured (OSN1), a majority of university students (61.3%) wore masks outdoors if they could not keep a safe distance away; for OSN2, 88.3% of participants kept their masks on indoors with non-cohabitants; for OSN3, 78.1% thought that meeting many people indoors during COVID-19 posed a risk; for OSN4, 75.7% avoided crowded indoor places during COVID-19; for OSN5, 76.1% of participants put on a mask when traveling by car with non-cohabitants; for OSN6, 91.4% sat on the terrace of bars, avoiding the interiors; for OSN7, 92% made sure that no one drank from their glass when they went out; and for OSN8, 78.8% of the respondents made sure that they did not share a plate when going out to eat. The data obtained were used to design the instrument using the Genially software programme and subsequently to implement the social norms campaign in the experimental study, which was distributed through WhatsApp.

#### Second part of the study: implementing the social norms campaign

We designed an experimental pre-test-post-test longitudinal panel study to explore the effectiveness of the social norms campaign designed to promote protective behaviours in university students. Once the campaign was designed, the 150 potential participants who were randomly assigned to the second part of the study were then randomly assigned to the experimental or control group for the experimental pre-test-post-test portion of the study. This random assignment of participants to each condition was done using the random sample of cases option of the SPSS’ select cases commando.

Considering that the experimental group would be exposed to an intervention for approximately 4 months, with four different phases, and then would probably suffer a higher experimental attrition, the randomisation programme assigned 100 participants to the experimental group and 50 to the control group. In total, 141 students participated in the first evaluation of the experimental pre-test-post-test portion of the study (95 from the experimental group and 46 from the control group), and 83 participants completed the last questionnaire (51 from the experimental group and 32 from the control group).^1^

**Figure 1:**
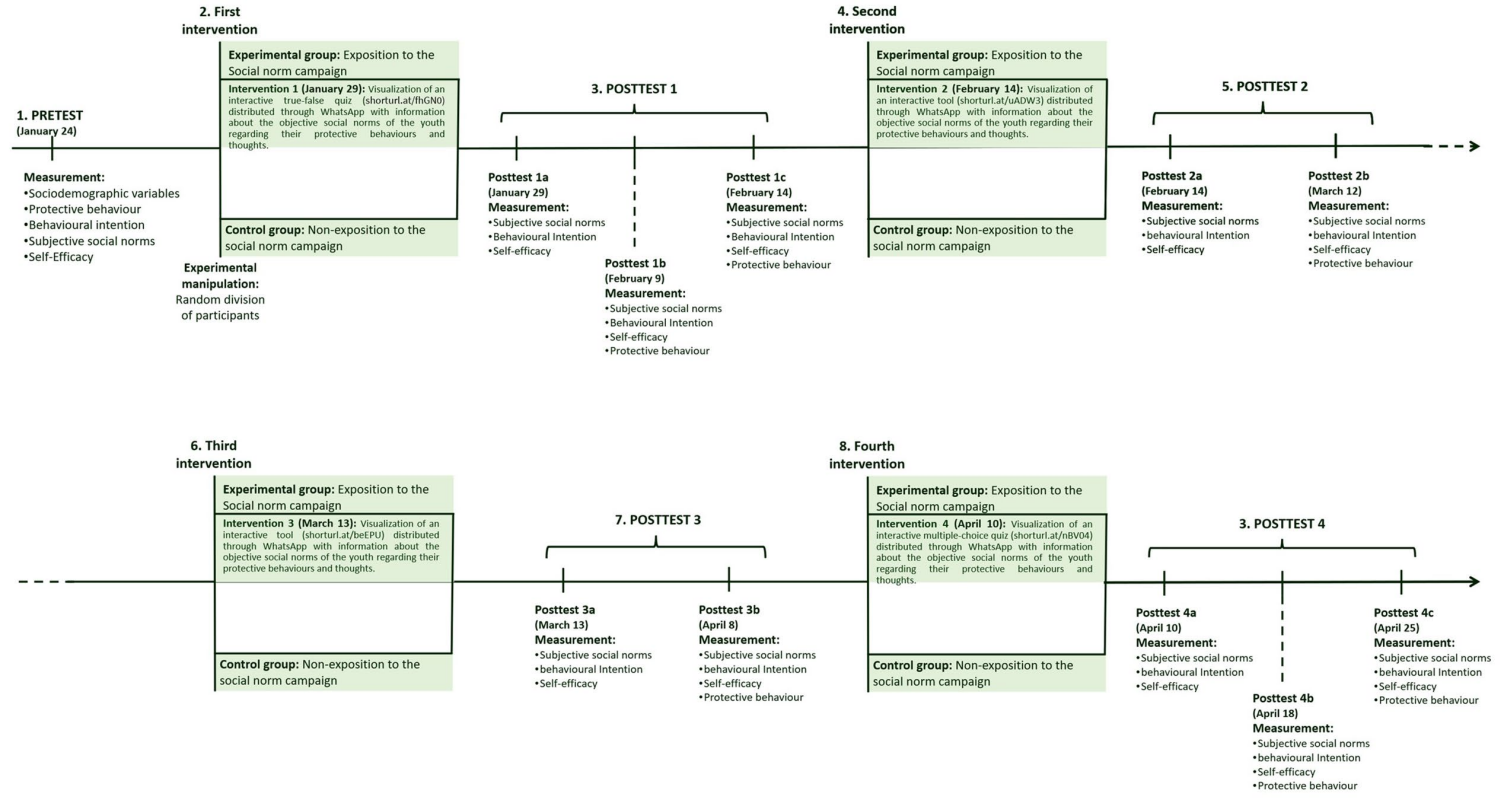
Diagram of the phases of intervention in the study.

As can be seen in Figure 1, the campaign was composed of four different phases with their corresponding intermediate and final post-test measures. The experimental group was exposed on four successive occasions, 1 month apart, to different interactive tools (weblinks to each original Spanish language tool) are available in Figure 1 and at:

First tool: https://view.genially.com/61a6abde789be10dd22be2b7
Second tool: https://view.genially.com/61ffb434da38a000186d7683
Third tool: https://view.genially.com/622924d57d159b001176a83e
Fourth tool: https://view.genially.com/6240a3f2f2692b001900a804

**Figure 2:**
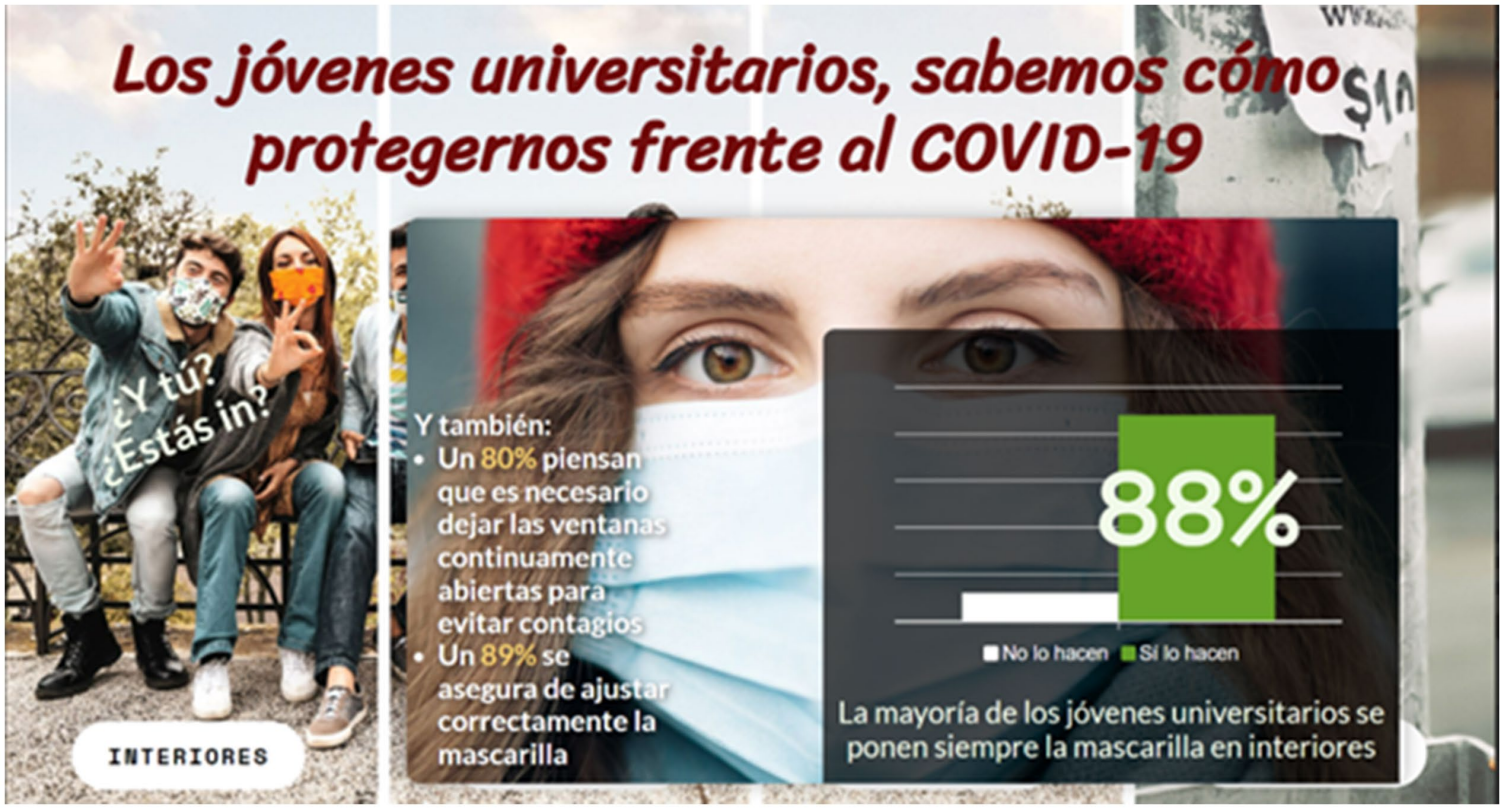
Sample of the last tool where one of the objective social norms can be observed. All the instruments of the social norms campaign are original creations that the author Esther Cuadrado has designed using the Genially tool (https://genially.com/es/). The images used in those instruments were downloaded by subscription to Freepick (www.freepik.es).

The control group was not exposed to any intervention. In each interactive tool created with Genially, participants in the experimental group were exposed to their reference group’s objective positive social norms regarding COVID-19 protective behaviour. As can be seen in both Figure 1 and the weblinks to the interactive tools, the first and last tools were interactive quizzes with information about the young people’s objective social norms, whilst the second and third tools were interactive instruments in which participants had to click on the pages to be informed of the objective social norms of their reference group. A sample of the second tool is offered in Figure 2. The instruments of the campaigns were designed by considering the key characteristics of the social norm campaign (32) and following the *guide to marketing social norms for health promotion in schools and communities* (33) and the National Social Norm Center resources at: https://socialnorms.org/.

The reason for using four different phases with different tools is that social norms campaigns need time and different instruments to be effective (32, 34). The impact of the campaign on subjective social norms, perceived self-efficacy to perform protective behaviours, protective behavioural intention, and protective behaviour itself was evaluated after each exposition to the intervention tool through an online questionnaire. To explore the changes in the study variables, participants of both the experimental and control groups completed the same questionnaire at the same time in each measurement time. The participation of the control group was limited to completing those questionnaires at each timepoint.

This study was approved by the Ethics Committee of the University of Córdoba (Ref. CEIH-22-4). Informed consent was obtained from each participant before the start of the study.

### Participants

In the first part of the study, to ascertain the objective social norm of the reference group, 749 university students (82.4% women, 17.1% men, 0.5% self-identified as fluid gender; mean age = 20.89, age range: 17–37 years, *SD* = 2.92) completed the questionnaire.

For the second part of the study, the experimental pre-test-post-test portion, the sample consisted of 141 univers ity students (83.1% female, 16.9% male) with a mean age of 21.55 years (range: 17–45, *SD* = 4.33), the control group consisting of 46 participants (87% female, 13% male) with a mean age of 21.30 years (range: 17–45, *SD* = 4.29), the experimental group consisting of 95 individuals (82.1% women, 17.9% men) with a mean age of 21.63 years (range: 17–45, *SD* = 4.36) at the beginning of the experiment. At the end of the experiment, 83 participants remained (81.9% female, 18.1% male; mean age = 22.12 years, *SD* = 5.29): 32 in the control group and 51 in the experimental group. The experimental attrition rates were 30.43 and 46.32% for the control and experimental groups, respectively. Assignment to each group was randomised; participation was voluntary and anonymous.

### Instruments

#### Subjective social norms

Subjective social norms to evaluate participants’ perception of their reference group thinking and behaviour in relation to anti-COVID-19 measures, a scale composed of eight items was specifically designed for the study objectives (Table 1) based on the objective social norms observed and analysed in the preliminary questionnaire. On a Likert-type scale from 1 to 5, participants said what percentage of students they thought performed the requested protective behaviours, where 1 indicated that the respondent thought that 0–19% (none or almost none) performed a certain behaviour and 5 meant the perception was 81–100% (all or almost all). The scale showed adequate reliability at each moment assessed (*α* = [0.84, 0.96]).

**Table 1:** Study scales.

Subjective social norms scale
**What percentage of young students do you believe think and act as described below in relation to measures to prevent COVID?**
1. Outdoors they put on a mask if they cannot keep a safe distance away.
2. Indoors they keep a mask on in the presence of non-cohabitants.
3. They think that meeting with many people indoors is a risk.
4. They avoid crowded indoor places.
5. They wear masks when driving with non-cohabitants.
6. They avoid sitting indoors in bars, preferring to sit outdoors.
7. They make sure not to share a cup/bottle when they go out.
8. They make sure not to share a plate when going out.
Self-efficacy for the adoption of protective behaviours scale
**To what extent do you feel capable of carrying out the following behaviours?**
1. Wearing a mask in the presence of other people outdoors.
2. Meeting with non-cohabitants only outdoors.
3. Avoiding crowded indoor places.
4. Avoiding going to parties, drinking parties and events with many people.
5. In restaurants and bars, removing the mask only when eating or drinking.
6. Sitting outside when going to bars or restaurants.
7. Making sure no one drinks from your glass when you go out.
8. Making sure you do not share your plate with anyone when you go out.
9. Keeping windows open when sharing a closed space with non-cohabitants.
10. Filtering the air when you share a closed space with non-cohabitants.
11. Performing some type of test before attending parties and events and not attending if you test positive.
12. Performing some type of test before meeting vulnerable people and not going if you test positive.
Intention to engage in protective behaviours scale
**How often do you intend to perform the behaviours described in the next 7 days?**
1. Outdoors, I will keep the mask on as long as I cannot maintain a safety distance of two feet from people I do not usually live with.
2. As much as possible, I will try to avoid crowded indoor spaces.
3. If I have to meet with people I do not live with, I will try to do it outdoors.
4. I will try to maintain two feet social distance from people I do not usually live with.
5. I will try to keep the windows open continuously whilst sharing interior space with non-cohabitants.
6. I will try to filter the air when I am in an indoor space shared with people I do not live with.
7. In the hypothetical case of going out for a drink in a bar/restaurant, I will sit outside.
8. In the hypothetical case of going out for a drink, I will make sure that no one drinks from my glass.
9. In the hypothetical case of sharing a car with people who do not usually live with me, I will wear a mask.
Protective behaviours scale
**How often have you done the behaviours described below in the past 7 days?**
1. Indoors, when I have been with people I do not usually live with, I have left the windows open continuously, even if not wide open, to maintain continuous ventilation.
2. I have kept safe distances from non-cohabitants.
3. Outdoors, if I could not maintain the safe distance of two feet from another person with whom I do not usually live, I have put on a mask.
4. I have avoided meeting indoors with my work groups and/or colleagues.
5. I have avoided attending events with crowds, even outdoors.
6. I have attended very crowded places and/or indoor events (reversed).

#### Self-efficacy for the adoption of protective behaviours

To assess the extent to which participants felt capable of adopting protective behaviour, we designed a scale specifically for the study (Table 1) following Bandura (25) self-efficacy scale construction guide. Participants answered the extent to which they felt able to perform 12 protective behaviours on a Likert-type scale from 1 to 5 (1 = not at all able, 5 = very able). The scale showed adequate reliability in each of the study phases (*α* = [0.84, 0.95]).

#### Intention to engage in protective behaviours

To measure participants’ intention to perform protective behaviours against COVID-19, a nine-item instrument (Table 1) was specifically designed for the study. Participants indicated the extent to which they intended to perform the described behaviours in the next 7 days on a five-point Likert-type scale (1 = never, 5 = always). The scale showed adequate reliability in each of the study phases (*α* = [0.89, 0.97]).

#### Protective behaviours

To evaluate the extent to which participants engaged in COVID-19 protective behaviours, a six-item scale was specifically created for the study (Table 1). Participants used a five-point Likert-type scale (1 = never, 5 = always) to report the extent to which they had performed the described behaviours in the past 7 days. The scale showed good reliability in each of the study phases (*α* = [0.75, 0.90]).

### Statistical analysis

In the present study, a mean comparison analysis was carried out between the control and experimental groups using Student’s *t*-test for independent samples. To explore whether there were significant differences between both groups after exposure to the Social Norms Campaign, 10 different t tests were carried out (one for each time these variables were evaluated) for each of the following three variables: subjective social norms, self-efficacy, and behavioural intentions; and six different *t*-tests were performed for the protective behaviour variable (one for each time this variable was evaluated).

Considering participant drop out, all the participants that took part in the phases for which the analysis was performed were included in each analysis. Thus, the number of participants who completed each phase of the study and were included in the analyses were all 141 participants in the pre-test and post-test 1a; 139 in the post-test 1b (93 and 46 in the experimental and control groups, respectively); 138 in the post-test 1c and the post-test 2a (93 and 45 in the experimental and control groups, respectively); 99 in the post-test 2b (64 and 35 in the experimental and control groups, respectively); 96 in the post-test 3a (61 and 35 in the experimental and control groups, respectively); 84 in the post-test 3b (51and 33 in the experimental and control groups, respectively); and 83 in the last three phases (51 and 32 in the experimental and control groups, respectively).

## Results

### Experimental manipulation effects: differences in subjective social norms between groups

**Figure 3:**
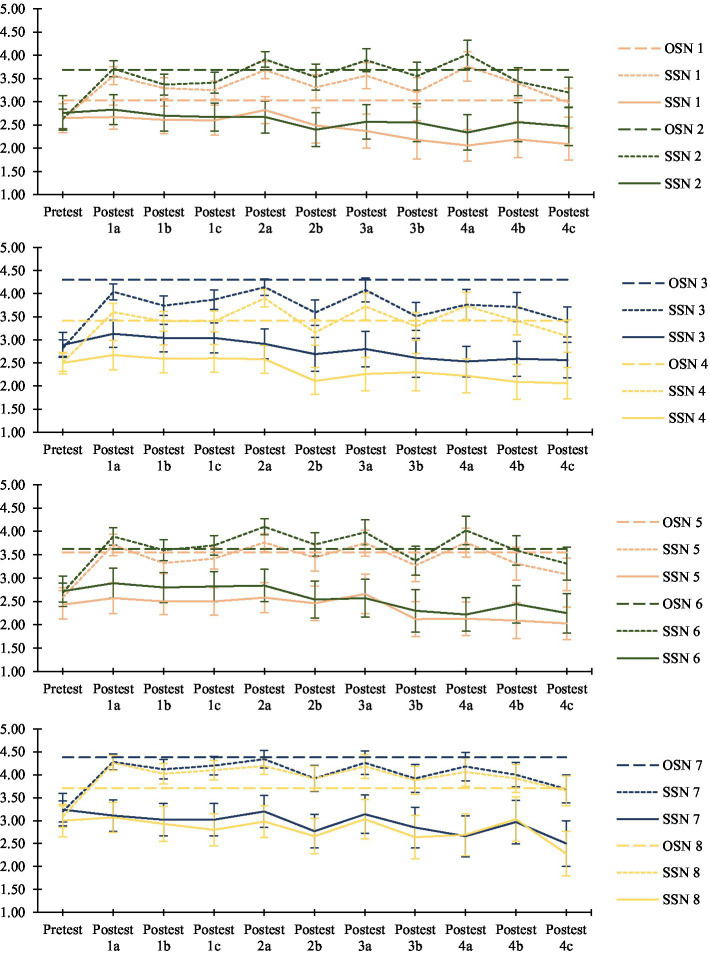
Results of the experimental manipulation on the perception of subjective social norms in the experimental group and the control group. OSN, objective social norm; SSN, subjective social norm; EG, experimental group; CG, control group. Each sub-figure refers to two different social norms. SN1, Outdoors they put on a mask if they cannot keep a safe distance away; SN2, Indoors they keep a mask on in the presence of non-cohabitants; SN3, They think that meeting with many people indoors is a risk; SN4, They avoid crowded indoor places; SN5, They wear masks when driving with non-cohabitants; SSN6, They avoid sitting indoors in bars, preferring to sit outdoors; SN7, They make sure not to share a cup/bottle when they go out; SN8, They make sure not to share a plate when going out.

As seen in Figure 3, Student’s *t*-test analyses showed no significant differences between the experimental group and the control group in the pre-test phase in any of the subjective social norms (SSN) evaluated: *t*_SSN1_ (*df* = 139) = −0.110, *p* = 0.912; *t*_SSN2_ (*df* = 74.58) = −0.594, *p* = 0.554; *t*_SSN3_ (*df* = 139) = −0.424, *p* = 0.672; *t*_SSN4_ (*df* = 103.56) = 0.34, *p* = 0.973; *t*_SSN5_ (*df* = 139) = 0.851, *p* = 0.396; *t*_SSN6_ (*df* = 139) = −0.121, *p* = 0.904; *t*_SSN7_ (*df* = 139) = −0.181, *p* = 0.853; *t*_SSN8_ (*df* = 139) = 0.451, *p* = 0.653. Before the intervention, therefore, control group and experimental group participants had similar perceptions of how their peers thought and behaved in terms of protective behaviours. However, as can be seen in Figure 3 and Table 2, in the different post-test phases, significant differences were found between the experimental group and the control group in all the subjective social norms evaluated, the experimental group showing higher^2^ levels of subjective social norms compared to the control group, levels that were closer to the objective social norms transmitted to them during the intervention. Therefore, after the intervention, participants in the experimental group were closer to reality in their estimates of what young university students thought and how they behaved in terms of protective behaviours. Thus, we can confirm that the experimental manipulation had the expected effect, allowing students to be more aware of what the objective social norm of their reference group is in terms of protective behaviours against COVID-19.

**Table 2:** *T*-test for independent samples of the experimental and control groups on social norms, self-efficacy, behavioural intention, and behaviour.

	Post-test 1a			Post-test 1b			Post-test 1c			Post-test 2a			Post-test 2b		
Variables	*t*	*df*	*p*-value	*t*	*df*	*p*-value	*t*	*df*	*p*-value	*t*	*df*	*p*-value	*t*	*df*	*p*-value
SSN1	5.263	139	<0.001	3.612	137	<0.001	3.563	136	0.001	5.037	136	<0.001	3.5	97	0.001
SSN2	5.175	139	<0.001	3.404	137	0.001	3.884	136	<0.001	7.214	136	<0.001	4.831	97	<0.001
SSN3	5.531	139	<0.001	3.732	137	<0.001	4.358	136	<0.001	7.12	136	<0.001	3.868	97	<0.001
SSN4	5.195	139	<0.001	4.225	137	<0.001	4.176	136	<0.001	7.55	136	<0.001	4.698	97	<0.001
SSN5	5.624	139	<0.001	4.225	137	<0.001	4.839	136	<0.001	6.535	136	<0.001	4.053	97	<0.001
SSN6	5.66	139	<0.001	4.065	137	<0.001	4.626	136	<0.001	6.885	136	<0.001	5.123	97	<0.001
SSN7	6.773	139	<0.001	5.515	137	<0.001	5.982	136	<0.001	6.162	136	<0.001	4.787	97	< 001
SSN8	7.201	139	<0.001	5.191	137	<0.001	6.481	136	<0.001	6.809	136	<0.001	5.353	97	<0.001
Self-efficacy	2.563	139	0.011	1.495	68,16	0.140	1.237	69.36	0.220	2.505	53.80	0.015	2.055	53.80	0.007
Intention	2.472	139	0.015	2.508	137	0.013	1.869	136	0.064	3.230	136	0.002	2.913	97	0.004
Behaviour	–	–	–	0.863	137	0.389	2.064	136	0.041	–	–	–	3.158	97	0.002
	Post-test 3a			Post-test 3b			Post-test 4a			Post-test 4b			Post-test 4c		
	*t*	*df*	*p*-value	*t*	*df*	*p*-value	*t*	*df*	*p*-value	*t*	*df*	*p*-value	*t*	*df*	*p*-value
SSN1	5.052	94	<0.001	3.868	82	<0.001	6.896	81	<0.001	4.736	81	<0.001	3.655	81	<0.001
SSN2	5.984	94	<0.001	3.936	82	<0.001	6.819	81	<0.001	3.394	81	0.001	2.718	81	0.008
SSN3	5.584	94	<0.001	3.483	82	0.001	4.944	81	<0.001	4.388	81	<0.001	3.186	81	0.002
SSN4	6.138	94	<0.001	3.986	82	<0.001	6.282	81	<0.001	5.452	81	<0.001	3.873	81	<0.001
SSN5	4.447	94	<0.001	4.346	82	<0.001	6.655	81	<0.001	4.488	81	<0.001	3.937	81	<0.001
SSN6	5.905	94	<0.001	3.943	82	<0.001	7.318	81	<0.001	4.414	81	<0.001	3.738	81	<0.001
SSN7	4.75	94	<0.001	4.148	82	<0.001	5.667	81	<0.001	4.017	81	<0.001	4.201	81	<0.001
SSN8	4.808	94	< 001	4.555	82	<0.001	5.044	81	<0.001	3.235	81	0.002	4.778	81	<0.001
Self-efficacy	3.121	49.69	0.003	2.718	51.50	0.009	2.707	48.19	0.009	2.724	51.45	0.009	2.087	81	0.040
Intention	3.838	55.44	<0.001	2.784	51.27	0.008	3.331	51.17	0.002	2.374	81	0.020	2.789	81	0.007
Behaviour	–	–	–	3.015	82	0.003	–	–	–	2.172	81	0.033	2.031	81	0.046

SSN, subjective social norm; SSN1, Outdoors they put on a mask if they cannot keep a safe distance away; SSN2, Indoors they keep a mask on in the presence of non-cohabitants; SSN3, They think that meeting with many people indoors is a risk; SSN4, They avoid crowded indoor places; SSN5, They wear masks when driving with non-cohabitants; SSN6, They avoid sitting indoors in bars, preferring to sit outdoors; SSN7, They make sure not to share a cup/bottle when they go out; SSN8, They make sure not to share a plate when going out.

### Perceived self-efficacy: differences between the experimental and control groups

**Figure 4:**
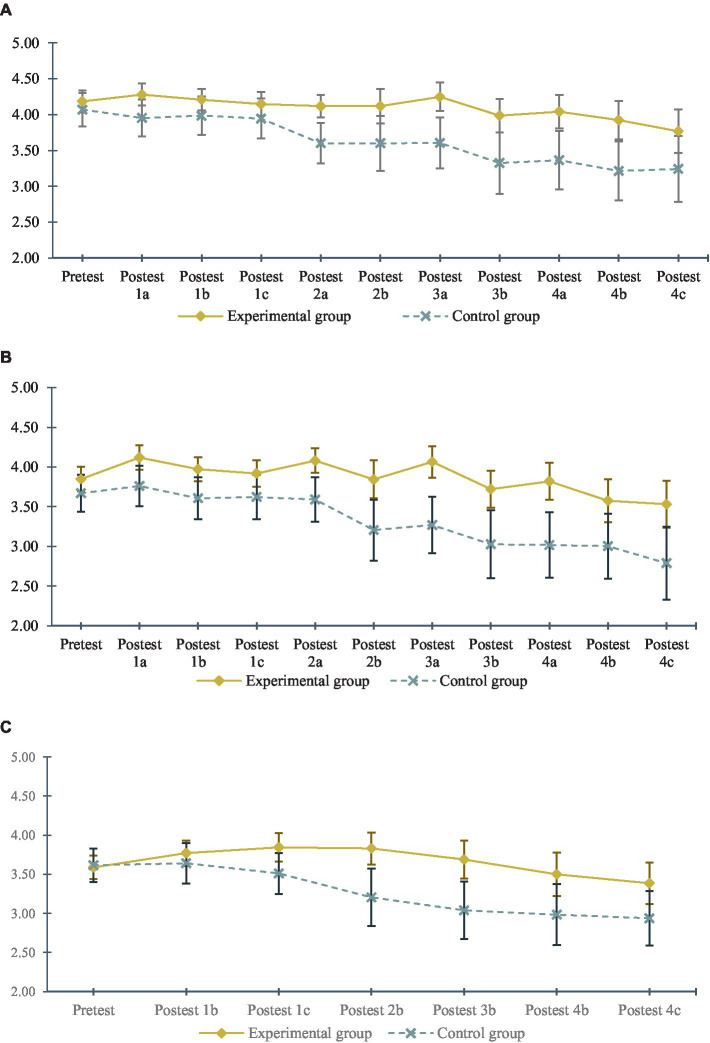
Evolution of variables in the control and experimental groups. **(A)** Evolution of self-efficacy in the control and experimental groups. **(B)** Evolution of behavioural intention in the control and experimental groups. **(C)** Evolution of protective behaviour in the control and experimental groups.

As shown in Figure 4A and Table 1, the analyses performed using Student’s *t*-test for independent samples showed that, although there were no significant differences between the groups in the pre-test, *t* (*df* = 139) = 1.040, *p* = 0.300 or in post-test 1b or post-test 1c, statistically significant differences were found in the rest of the post-tests.^3^ Hypothesis 1b was thus confirmed: exposure to a social norms campaign applied to university students’ protective behaviours increases their levels of self-efficacy for protective behaviours.

### Behavioural intention: differences between the experimental and control groups

As shown in Figure 4B and Table 1, the analyses performed using Student’s *t*-test for independent samples showed no significant differences between the groups in the pre-test, *t* (*df* = 139) = 1.274, *p* = 0.205 or in post-test 1c (whose difference was marginal at *p* = 0.064); however, statistically significant differences were found in the remaining post-tests.^4^ Hypothesis 1a was thus confirmed: Exposure to a social norms campaign applied to university students’ protective behaviours increases their levels of intention for protective behaviours.

### Behaviour: differences between the experimental and control groups

As shown in Figure 4C and Table 1, the analyses performed using Student’s *t*-test for independent samples showed no significant differences between the groups in the pre-test, *t* (*df* = 139) = −0.211, *p* = 0.833, or the post-test 1b; however, statistically significant differences were found in the remaining post-tests.^5^ Hypothesis 1c was thus confirmed: Exposure to a social norms campaign applied to university students’ protective behaviours increases their levels of protective behaviours against COVID-19.

## Discussion

The aim of this study was to explore the effectiveness of a social norms campaign applied to COVID-19 protection behaviours among university students. To this end, a social norms campaign was designed using interactive tools distributed through WhatsApp. This campaign introduced participants to the positive social norms of university students, most of whom adopted protective behaviours during the pandemic. Positive social norms reported were of the type “The majority (88%) of university students always wear their masks indoors” or “92% of university students make sure no one drinks from their glass when they go out,” among others. The experimental group was exposed on four successive occasions 1 month apart to different interactive tools. After each intervention, the impact of the campaign on subjective social norms, perceived self-efficacy to perform protective behaviours, protective behavioural intention, and protective behaviour itself was evaluated.

The results found are promising, as they confirm the potential efficacy of social norms campaigns for the promotion of protective behaviours in undergraduate students in times of pandemic. Through their focused theory of normative behaviour, Cialdini et al. (9) highlight the importance of social norms in the regulation of human behaviour, suggesting that behaviours are more likely to be influenced by norms that are the focus of attention in a specific context—in this case, the context of the pandemic, normative behaviours referring to protective behaviours against the disease. In this line, the present study has shown how the application of descriptive social norms through campaigns that inform and make them visible is useful and influences the regulation of behaviour. In fact, there is evidence (15, 17, 18, 20) that descriptive norms (information about the behaviour of the majority of people of a certain group) significantly influence behaviour regulation. Authors such as Lally et al. (17) and Mollen et al. (19) report these same effects on behaviour in adolescent and young adult populations. Thus, the application of social norms has been a useful and effective tool at the level of intervention in health behaviours (21).

This study demonstrated that when university students were exposed to their reference group’s positive social norms through a social norms campaign in which they were shown that most university students adopted protective behaviours, they espoused a greater intention to adopt such behaviours, confirming H1a; their self-efficacy to perform protective behaviour increased, confirming H1b; and they adopted more protective behaviours, confirming H1c.

The first result obtained was confirmation of the effectiveness of the experimental manipulation. The social norms campaign implemented proved to be effective in modifying the subjective social norm of the students, that is, their perception of the social norm of their reference group. Thus, after exposure to the campaign, the participants showed that they had a more realistic knowledge of the protective behaviours implemented by their peer group against COVID-19. This first result is fundamental, not only because it shows the effectiveness of the experimental manipulation and the social norms campaign implemented, but because it represents a first step for possible behavioural change, given that scientific literature has shown that the subjective social norm of individuals is a strong predictor of their behavioural intention and behaviour (7–13).

In relation to behavioural intention, the results obtained in the present study are in line with previous research [e.g., (10, 36)] that demonstrated the effect of objective social norm exposure on behavioural intentions in different domains. The TPB (8) describes how behavioural attitudes, subjective social norms, and behavioural control influence people’s behavioural intentions, which in turn influence the enactment of a given behaviour (14). In this vein, our study has shown that the implementation of a social norms campaign can positively influence the behavioural intention of university students, resulting in an increase in this variable compared to a control group: participants adjusted their intention to adopt protective behaviours to the social norms to which they were exposed in the social norms campaign.

Additionally, in line with Bandura (25), this study posited that given that the reference group functions as a model, applying a social norms campaign in which individuals are shown that their reference group mostly performs a certain behaviour will function as modelling by influencing people’s levels of self-efficacy. The results obtained study support this hypothesis, since a higher level of self-efficacy for the adoption of protective behaviours was found in those participants to whom the social norms campaign was applied, whilst the group that did not receive this intervention did not show any change, confirming H1b. Therefore, the application of a social norms campaign was effective in increasing self-efficacy: students exposed to the social norms campaign felt more capable of adopting protective behaviours than those who were not exposed to the campaign. From a theoretical point of view, this result is especially relevant if it is considered that, as far as we know, no previous studies have analysed the relationship between social norms and self-efficacy. Nevertheless, the theoretical background supports this hypothesis, which this study has now confirmed.

Finally, it was observed that the participants in the experimental group (but not those in the control group) positively improved their protection behaviours against COVID-19 when they were exposed to the objective social norms of their peer group. These results fit with studies by Cuadrado et al. (7) and Cuello Díaz (37) in which positive subjective social norms were shown to influence the adoption and maintenance of protective behaviours during the pandemic.

The results obtained in the present study are of relevance at the practical level. The results highlight how the application of a social norms campaign effectively increases not only young people’s intention to adopt protective behaviours and their self-efficacy in a pandemic context, but also their protective behaviour. This shows the effectiveness of such campaigns, simple to apply and low cost, in the promotion of protective behaviours related to health issues. In this sense, the present study addresses a critical aspect of public health response. In times of global health emergencies, understanding and influencing human behaviour becomes paramount. This research not only contributes valuable insights into behavioural science but also offers practical implications for enhancing public health strategies, and provides a framework for developing effective interventions that can mitigate the spread of infectious diseases and protect communities worldwide.

### Limitations and future lines of research

Inevitably, this study has certain limitations. Among them, we must highlight the use of questionnaires as a method for data collection, which could generate social desirability bias. That is to say, participants could over-report positive behaviours or under-report negative behaviours. To control this limitation, we posed different questions that refer to the same behaviour in different ways. In future research, alternative methods of data collection are proposed to avoid this potential bias, such as asking questions that are as neutral as possible to avoid socially desirable responses or comparing self-reported measures with objective measures or external observations without the influence of individual interpretation.

Another limitation is the loss of participants as the study progressed. The initial sample included 141 participants but there were significant attrition rates, 30.43 and 46.32% for the control and experimental groups respectively, which could affect the validity of the results. This could have been due to the relatively long time of the study (3 months) with many repetitions of the questionnaires (11 questionnaires; one pre-test and 10 post-tests) to ensure adequate investigation, which is typical of longitudinal panel research. Nevertheless, participant attrition was minimised by offering as a reward the raffle of a tablet among those who completed all phases of the study. However, to mitigate the loss of participants in this type of longitudinal design, it is proposed to use larger sample sizes.

Another limitation refers is the lack of explicit criteria for inclusion and exclusion of participants and the use of non-probabilistic convenience sampling. Convenience sampling can introduce bias, as it may not represent the broader population. However, the large sample size (141 participants) and the focus on university students help mitigate this issue to some extent. Future research should use probabilistic and random sampling, and increase the geographic diversity and the sample size to increase the representativeness and generalisation of the results, obtain more evidence, and extend the study conclusions across more diverse groups. Similarly, future studies should include participants from diverse demographic backgrounds to examine the generalizability of the findings across various populations.

In addition, it would be interesting to control for confounding variables such as previous exposure to similar campaigns or different levels of knowledge—in this case, about COVID-19. This could be included as a measure prior to application of the questionnaire, as an exclusion criterion for participants who have been exposed to other campaigns or have certain levels of previous knowledge.

Likewise, a long-time data evaluation check to analyse whether protective behaviours were maintained over a long period was not conducted. Future studies could include long-term follow-up studies to assess the sustainability of the intervention effects over extended periods. However, given the specific context of the application, and considering that in July 2022 the pandemic situation had calmed down, it did not make sense to continue evaluating the effectiveness of our campaign since it was no longer as necessary to carry out the protective behaviours evaluated, such as wearing a mask or continuously ventilating enclosed spaces.

Furthermore, it would be interesting to obtain evidence of the effectiveness of our social norms campaign in different cultures, since, in the context where it was carried out, there were cultural aspects that probably had an influence, such as the type of social and leisure contacts. Moreover, it would be interesting to explore the applicability of the campaign in other epidemic contexts and its generalizability to other health emergency situations and in other health contexts.

Another fruitful line of research might involve carrying out a post-intervention study over a longer period, which would allow long-term monitoring to assess the sustainability of intervention effects over extended periods of time. Similarly, exploring the effects of different intensities and durations of social norms campaigns would be useful in determining the most effective approaches to behaviour change.

## Conclusion

This study has demonstrated that the application of a social norms campaign in a student population can lead to increases in behavioural intention, self-efficacy, and protective behaviours during a global pandemic. The research has demonstrated the potential effectiveness and some benefits of a social norms campaign in a pandemic situation. Our findings might be extrapolated to other health emergencies or health contexts.

Following our results, social norms campaigns focusing on protective behaviours could show promising results in promoting positive health outcomes beyond COVID-19. By highlighting the prevalence of desired behaviours within a community, such campaigns can effectively influence individuals to adopt similar actions. This approach can be applied in various real-world settings, such as promoting safe driving practises, encouraging regular exercise, or advocating sustainable lifestyle choices.

To build on the study’s results, additional research is needed in several key areas. Firstly, investigating the long-term sustainability of behaviour change induced by social norms campaigns is crucial to assess their lasting impact. Additionally, exploring the effectiveness of tailored messaging for different demographic groups can enhance the campaign’s reach and relevance. Furthermore, studying the influence of social networks and peer interactions on behaviour adoption can provide valuable insights for optimising future campaigns.

Overall, social norms campaigns offer a powerful tool for promoting protective behaviours in diverse contexts. Continued research in these areas can help refine strategies and maximise the impact of such campaigns in improving public health outcomes.

## Data Availability

The datasets presented in this study can be found in online repositories. The names of the repository/repositories and accession number(s) can be found at: Cuadrado (38).

## Footnotes

^1^No *a priori* test was used to decide the sample size. However, a *post-hoc* power analysis was performed by entering as statistical test “Means: Differences between two independent means (two groups)” from the “t tests” test family in GPower. The results showed that, given an error probability of *α* = 0.05, a medium effect size of *d* = 0.50, and the study sample at the starting point of the study (i.e., 95 participants in the experimental group and 46 in the control group), the power of the effect achieved is 0.86; with the same data but a medium to large effect size of *d* = 0.65, the power of effect is 0.97. When performing the same analysis with the final sample size (51 from the experimental group and 32 from the control group), the power of the effect reached 0.88 with a medium to large effect size of *d* = 0.65, and 0.97 with a large effect size of *d* = 0.80. So, the sample size can be considered sufficient at the starting point and acceptable at the end of the study.

^2^If applying the Bonferroni correction to the significance threshold, for the effect of experimental condition to be significant, its *p*-value would have to be less than 0.05/10, or 0.005 (35). When the more conservative Bonferroni method is followed, all the effects of the experimental condition were significant.

^3^If applying the Bonferroni correction to the significance threshold, for the effect of experimental condition to be significant, its *p*-value would have to be less than 0.05/10, or 0.005 (35). When the more conservative Bonferroni method is followed, only one effect of the experimental condition remains significant.

^4^If applying the Bonferroni correction to the significance threshold, for the effect of experimental condition to be significant, its *p*-value would have to be less than 0.05/10, or 0.005 (35). When the more conservative Bonferroni method is followed, five effects of the experimental condition were significant.

^5^If applying the Bonferroni correction to the significance threshold, for the effect of experimental condition to be significant, its *p*-value would have to be less than 0.05/6, or 0.008 (35). When the more conservative Bonferroni method is followed, only two effect of the experimental condition were significant.
