# Supplementary material for: A social norms campaign based positive intervention aimed at promoting protective behaviours
Source: Front Public Health. 2024 Oct 24;12:1447335. doi: 10.3389/fpubh.2024.1447335 (PMC11544322; doi:10.3389/fpubh.2024.1447335)
Supplement: Supplementary file 1 [file Table_1.DOCX]

**Supplementary File 1. Debriefing**

We want to thank you for participating in the study.

You have been part of a study in which **two random groups have been formed**. **In one of them, some interactive infographics have been sent about what university students think and do in relation to protection measures against COVID.** The data and information about what university students think and do was obtained thanks to the participation of about 750 students who responded to a survey on attitudes and behavior of young people carried out in December 2021. **The other group has not received such infographics.**

**The objective was to study whether having normative information about what the peer group does and thinks** in relation to protective behaviors **ultimately affects the intentions and behaviors of individuals**, causing them to adopt greater protective behaviors. This type of study is important to help promote protective behaviors in pandemic situations and especially in times when the adoption of protective behaviors is necessary to reduce disease transmission. In addition, it allows us to **study how social norms influence groups, in order to apply protection campaigns in other health areas**.

Once again, **we thank you** for your valuable participation, and **we wish you luck in the next tablet giveaway**.
